# Unleashing the potential: further enhancing the impact of Natural Products and Bioprospecting

**DOI:** 10.1007/s13659-023-00388-x

**Published:** 2023-08-17

**Authors:** Ji-Kai Liu, Xiao-dong Luo, Ling Zhang, Ying Zhang

**Affiliations:** 1 School of Pharmaceutical Science, South-Central University for Nationalities, Wuhan, 430074 People’s Republic of China; 2 School of Chemical Science and Technology, Yunnan University, Kunming, 650500 People’s Republic of China; 3 State Key Laboratory of Phytochemistry and Plant Resources in West China, Kunming Institute of Botany, Chinese Academy of Sciences, Kunming, 650201 People’s Republic of China; 4 Medicine & Life Sciences Journals, Springer Nature, Beijing, 100080 People’s Republic of China

Academic journals are vital for the dissemination of scientific knowledge, the encouragement of scholarly discussions, and the advancement of research across various disciplines. The impact of a journal, measured by its influence on academic and professional communities, is a key indicator of its success. In the latest Journal Citation Report (JCR) released by Clarivate in June 2023, *Natural Products and Bioprospecting* (NPB) got the first impact factor of 4.7. This is another milestone in the journal’s efforts to expand its academic influence. *Natural Products and Bioprospecting* is currently indexed by several prominent databases, including the Emerging Sources Citation Index (ESCI), the Directory of Open Access Journals (DOAJ), PubMed Central, and Scopus. The 2022 Citescore of the journal is 7.5, ranking at Q1 of Plant Science and Food Science, Q2 of Analytical Chemistry, Biochemistry, Organic Chemistry, Pharmacology, and Toxicology. The journal has a yearly downloads of more than 326k, and a yearly Altmetric mentions of over 350.

The question of ‘When NPB will have the first impact factor’ was often raised by research community since the journal was officially launched in 2011. It is generally recognized that the Impact Factor is one of key metrics in evaluating a journal’s quality and performance, however, the editorial team of NPB has been paying great attention to unlock journal’s potential from all angles.

Initially, to enhance the experience for authors, the editorial team and the publisher have made efforts towards reducing the turnaround time for peer review, editorial decisions, and production. In 2022, NPB’s median turnaround time from submission to first decision was 12 days, from submission to final accept decision was 49 days; the average production turnaround time was 18 days.

Secondly, the editorial team recognizes the significance of fostering diversity in both the submissions received and the published content. This includes not only diverse topics but also contributions from a wide range of geographic locations. Over 90% of the submissions and around 40% of the published content in 2022 were from outside of China.

Thirdly, the editorial team prioritizes the quality of each accepted article. According to Clarivate’s 2022 Journal Citation Report, more than 80% of NPB’s research articles and reviews published in 2020 and 2021 contributed citations to its 2022 Impact Factor. This notable statistic indicates that the quality of NPB’s published content is relatively average. However, it is worth mentioning the articles that have received high citation counts.

These are the 2022 Top 10 frequently cited NPB papers published in 2020 and 2021 (Table 1) elucidates bioactive compounds and reviews. For example, the most cited paper “Natural Products in Cancer Therapy: Past, Present and Future” by M. Huang, J. J. Lu, and J. Ding (*Nat. Prod. Bioprospect.* 2021, 11: 5–13) has been cited 61 times.

**Table 1:** The most cited articles in 2022 to articles published in 2020 and 2021

	Authors	Topic	Citations
1	Jian Ding et al. 2021	Natural Products in Cancer Therapy: Past, Present and Future	61
2	Rameshwar S. Cheke et al. 2020	Recognition of Natural Products as Potential Inhibitors of COVID-19 Main Protease (Mpro): In-Silico Evidences	39
3	Nagendra Singh Chauhan et al. 2021	Plants Used as Antihypertensive	21
4	Ilkay Erdogan Orhan et al. 2020	Natural Products as Potential Leads Against Coronaviruses: Could They be Encouraging Structural Models Against SARS-CoV-2?	20
4	Guanhua Du et al. 2020	Research Progress of the Antiviral Bioactivities of Natural Flavonoids	20
6	Ayman Khalil et al. 2020	The upshot of Polyphenolic compounds on immunity amid COVID-19 pandemic and other emerging communicable diseases: An appraisal	17
7	Takshma Bhatt et al. 2020	Carotenoids: Potent to Prevent Diseases Review	16
8	Pukar Khanal and B. M. Patil et al. 2020	Anthraquinone Derivatives as an Immune Booster and their Therapeutic Option Against COVID-19	15
8	Yue-Hu Wang 2021	Traditional Uses and Pharmacologically Active Constituents of Dendrobium Plants for Dermatological Disorders: A Review	15
10	Yang Lu et al. 2020	Anti-inflammatory Effects and Mechanisms of Rhein, an Anthraquinone Compound, and Its Applications in Treating Arthritis: A Review	13

Moreover, another significant and noteworthy aspect to consider is the proportion of self-citations. A high self-citation rate may indicate a limited scope within a discipline or raise concerns about potential citation manipulation. NPB promotes fair citation practices, leading to a low rate of self-citation of 2%. The journal’s impact factor without self cites was 4.6 in 2022 JCR year. This, we believe, was a very healthy indicator.

The traditional Chinese zodiac cycle is 12 years. In the past 12 years, through the joint efforts of authors, editorial board members, and readers from the global scientific community, as well as the publisher, we have successfully navigated numerous challenges in transforming NPB from a newly established journal into a highly valued and appealing communication platform for the natural products scientific community. As we look towards the future, we expect NPB to deliver an even more remarkable performance in the upcoming cycle, and we hope that more and more natural product scientists from around the world will publish their good research work and new academic ideas with NPB.

*Natural Products and Bioprospecting* (NPB) is a fully open access journal that sponsored and supported by Kunming Institute of Botany, Chinese Academy of Sciences, and published in partnership with Springer Nature. It serves as an international forum for essential research on the science of natural products. NPB publishes original research articles, reviews and short communications, aiming to rapidly disseminate research results of timely interest, and comprehensive reviews of emerging topics in all the areas of natural products. NPB adopts a continuous publishing model, which allows for the immediate publication of articles as soon as they are ready.

